# Layered metal sulfides with M_a_S_b_^c−^ framework (M = Sb, In, Sn) as ion exchangers for the removal of Cs(Ⅰ) and Sr(Ⅱ) from radioactive effluents: a review

**DOI:** 10.3389/fchem.2023.1292979

**Published:** 2023-12-06

**Authors:** Qi Zhao, Shuai Wang, Yichun Wu, Yixuan Wang, Shengshou Ma, Kaimin Shih

**Affiliations:** ^1^ Department of Civil Engineering, The University of Hong Kong, Pokfulam, Hong Kong SAR,China; ^2^ School of Metallurgy, Northeastern University, Shenyang, Liaoning, China

**Keywords:** layered metal sulfides, radioactive effluents, ion exchange, cesium, strontium

## Abstract

Nuclear power has emerged as a pivotal contributor to the global electricity supply owing to its high efficiency and low-carbon characteristics. However, the rapid expansion of the nuclear industry has resulted in the production of a significant amount of hazardous effluents that contain various radionuclides, such as ^137^Cs and ^90^Sr. Effectively removing ^137^Cs and ^90^Sr from radioactive effluents prior to discharge is a critical challenge. Layered metal sulfides exhibit significant potential as ion exchangers for the efficient uptake of Cs^+^ and Sr^2+^ from aqueous solutions owing to their open and exchangeable frameworks and the distinctive properties of their soft S^2−^ ligands. This review provides a detailed account of layered metal sulfides with M_a_S_b_^c−^ frameworks (M = Sb, In, Sn), including their synthesis methods, structural characteristics, and Cs^+^ and Sr^2+^ removal efficiencies. Furthermore, we highlight the advantages of layered metal sulfides, such as their relatively high ion exchange capacities, broad active pH ranges, and structural stability against acid and radiation, through a comparative evaluation with other conventional ion exchangers. Finally, we discuss the challenges regarding the practical application of layered metal sulfides in radionuclide scavenging.

## 1 Introduction

The 21st century has witnessed an unprecedented surge in global energy demand, driven by rapid population growth and industrial expansion (Hao et al., 2023). This increased demand has placed a burden on conventional fossil fuel resources, as has the growing emphasis on environmental protection, energy security, and global warming (Awual, 2016; Sun et al., 2019). In this context, nuclear electricity production has rapidly expanded globally and is expected to increase by 30% from 2017 to 2030 (Fawzy et al., 2020). Many countries have developed and expanded their nuclear energy strategies. In July 2022, the European Parliament approved labeling nuclear energy projects as “green,” paving the way for the construction of new reactors in the European Union (Stevis-Gridneff and Sengupta, 2022). Similarly, China aims to build at least 150 new reactors between 2021 and 2036 to reduce carbon emissions; this number is larger than the present total number of new nuclear reactors in the rest of the world (Murtaugh and Chia, 2021). However, significant amounts of radioactive effluents are generated during the operation and decommissioning of nuclear facilities (Rivasseau et al., 2013; Querfeld et al., 2019). The treatment and discharge of large amounts of radioactive liquid effluents, particularly following incidents such as the Fukushima Daiichi nuclear disaster, have become a primary societal concern (Castrillejo et al., 2016). Generally, the radioactive effluents from nuclear reactors contain dozens of fission products, among which ^137^Cs (*t*_1/2_ = 30 years) and ^90^Sr (*t*_1/2_ = 29 years) are particularly hazardous owing to their high yields, long half-life, high-energy β/γ emissions, and considerable water solubility (Steinhauser, 2014). These isotopes can easily accumulate in the human body through contaminated water and the food chain (Kim et al., 2021), posing severe health risks such as cancer, leukemia, and genetic disorders (Mangano and Sherman, 1961; Kamiya et al., 2015; Chen et al., 2020). Therefore, the effective elimination of radioactive Cs^+^ and Sr^2+^ is crucial for environmental safety and human health.

Various methods have been employed to remove Cs^+^ and Sr^2+^ from radioactive effluents before their release into the environment. These methods include evaporation (Ma et al., 2023), chemical precipitation (Lei et al., 2019), membrane separation (Hao et al., 2018), solvent extraction (Gerow et al., 1981), and ion exchange (Ding et al., 2013b). However, most of these techniques suffer from significant drawbacks, such as high energy consumption, low removal yield, membrane fouling, high operating costs, low selectivity, toxicity, the generation of a massive amount of secondary waste, and process instability under acidity and radiation (Wang and Zhuang, 2019). Among these, ion exchange is widely regarded as the most promising solution owing to its high efficiency, ease of operation, low cost, and minimal secondary waste (Marinin and Brown, 2000; Nilchi et al., 2011; Alby et al., 2018). Ion exchange also plays a central role in the treatment of nuclear wastewater from the Fukushima Daiichi nuclear disaster (Lehto et al., 2019). Thus, the development of suitable ion exchange materials for efficiently removing Cs^+^ and Sr^2+^ from contaminated effluents is a critical challenge worldwide.

Conventional ion exchange materials are characterized by limited effectiveness in treating radioactive effluents. Common ion exchange materials, such as clays (El-Dessouky et al., 2018) and zeolites (Merceille et al., 2012; Mahima Kumar et al., 2021), are unstable under relatively acidic conditions, while titanates suffer from poor selectivity in the presence of high acidity or salt concentrations (Ding et al., 2013a; Zhao et al., 2018). Furthermore, exposure to radiation during the treatment of radioactive liquids can damage the structure of these materials and affect their performance (Zhao et al., 2021; Zhao et al., 2022a). In recent years, layered metal sulfides have emerged as a promising class of ion exchangers (Ding and Kanatzidis, 2010). These materials possess open-layered frameworks, which enable the ion exchange of radionuclides with interlayer cations within the internal structure of layered metal sulfides (Zhang et al., 2021). According to Pearson’s hard–soft acid–base theory (Pearson, 1966; Parr and Pearson, 1983), hard acids prefer hard bases and soft acids prefer soft bases, both thermodynamically and kinetically. The S^2−^ ligands in layered metal sulfides are considered soft bases due to their high polarizability. Consequently, they exhibit a preference for interacting with soft or moderately soft acids (Ding and Kanatzidis, 2007), such as Cs^+^ and Sr^2+^ (Pearson, 1963). However, the common coexisting ions in aqueous environment, e.g., H^+^, Na^+^, K^+^, Ca^2+^, and Mg^2+^, belong to the hard cation family and display weak interactions with the soft S^2−^ ligands (Kromah and Zhang, 2021). This difference in complexation preference of the S^2−^ ligand framework endows a special selectivity to layered metal sulfides in the removal of Cs^+^ and Sr^2+^ from effluent (Chen et al., 2020). Additionally, the stable metal–sulfur bonds of layered metal sulfides provide them with resistance to acidity and radiation, which makes them superior to conventional oxide and organic ion exchangers for treating radioactive effluents (Alby et al., 2018).

Despite their promising properties for metal removal, research on layered metal sulfides as ion exchangers is still in the early stages, with fewer than 30 compounds reported to date (Qi et al., 2015). In particular, there is a lack of comprehensive reviews of the application of layered metal sulfides in the removal of Cs^+^ and Sr^2+^. Hence, this article aims to provide an overview of layered metal sulfides with a M_a_S_b_^c−^ framework (M = Sb, In, Sn), focusing on their synthesis routes, structural features, and removal properties of Cs^+^ and Sr^2+^. Finally, we identify challenges and future research areas related to layered metal sulfides and highlight their potential for practical applications in the treatment of radioactive liquid waste. This article focuses on the layered metal sulfides with a M_a_S_b_^c−^ framework (M = Sb, In, Sn). Other variations, such as layered metal sulfides in which divalent metals (e.g., Mg^2+^, Mn^2+^, Zn^2+^) partially replacing the framework metal, such as K_2x_Mn_x_Sn_3−x_S_6_ (Manos and Kanatzidis, 2009), K_2x_Mg_x_Sn_3−x_S_6_ (Mertz et al., 2013), and Na_5_Zn_3.5_Sn_3.5_S_13_·6H_2_O (Zhang et al., 2020), are not discussed.

## 2 Synthesis methods

The hydrothermal and solvothermal methods are the most common routes for preparing layered metal sulfides (Sarma et al., 2016). These approaches allow the controlled synthesis of layered metal sulfides with specific structural properties by enabling the reaction of appropriate metals and sulfur in an aqueous or solvent environment at high temperatures and pressures. The hydrothermal process typically involves the following steps. Metal sources (pure metals or metal salts) and sulfur are weighed to achieve an appropriate mass ratio. The mixture is then placed in a stainless-steel autoclave. Deionized water is added dropwise until a dough-like consistency forms. The autoclave is sealed and heated in a thermostatically controlled oven. The temperature and duration of the heating process vary depending on the desired product. After the heating process, the autoclave is cooled to room temperature. The product is separated from the reaction mixture through centrifugation and filtration. The isolated material is washed several times with deionized water and organic solvents (such as ethanol and acetone) to remove impurities. The washed material is vacuum-dried to eliminate the remaining solvent or moisture. Finally, the dried layered metal sulfides are obtained through grinding. The solvothermal method follows the same general steps. The only difference is that organic solvents are used as the reaction medium instead of water. The organic solvents are likely to participate in the synthesis reaction and act as the interlayer cations for space filling and charge compensation. This enables the construction of complex architectures that may not be achieved in aqueous media (Wang et al., 2016). Table 1 summarizes the synthesis conditions for the layered metal sulfides discussed in this paper.

**TABLE 1:** Summary of synthesis conditions of the layered metal sulfides included in the study.

Crystal	Reactant	Reaction media	Temp. (°C)	Time (days)	Yield	Ref.
SbS-1	Sb, S, N_2_H_4_·H_2_O	DES^a^	180	1	86.5%	Zhao et al. (2022b)
FJSM-SbS	Sb, S, CH_3_NH_2_	Ethanol	160	7	62.0%	Liao et al. (2021)
InS-1	InCl_3_, Trithiocyanuric acid	Ethylamine ethanol	160	4	N/A^b^	Wang et al. (2020)
InS-2	In, S	Ethylamine ethanol	140	4	54.0%	Sun et al. (2020).
NaTS	Na_2_CO_3_, Sn, S	Deionized water	120	1	N/A	Zhang et al. (2019b)
FJSM-SnS	SnCl_4_·5H_2_O, S	Dimethylamine	180	7	80.4%	Qi et al. (2015)
FJSM-SnS-2	SnCl_4_·5H_2_O, S	[Bmmim]Cl^c^ + methylamine	180	5	65.8%	Li et al. (2021b)
FJSM-SnS-3	Sn, S	[Bmmim]Cl + methylamine	180	5	57.7%	Li et al. (2021b)
FJSM-SnS-4	SnCl_4_·5H_2_O, S	Ethylamine	180	7	43.7%	Li et al. (2021a)
KTS-3	K_2_CO_3_, Sn, S	Deionized water	220	0.6	85.0%	Sarma et al. (2016)

^a^ DES: deep eutectic solvent composed of isopropylamine hydrochloride and urea.

^b^ N/A: not available.

^c^ [Bmmim]Cl: 1-butyl-2, 3-dimethylimidazolium chloride.

## 3 Layered metal sulfides for Cs^+^ and Sr^2+^ removal

**FIGURE 1:**
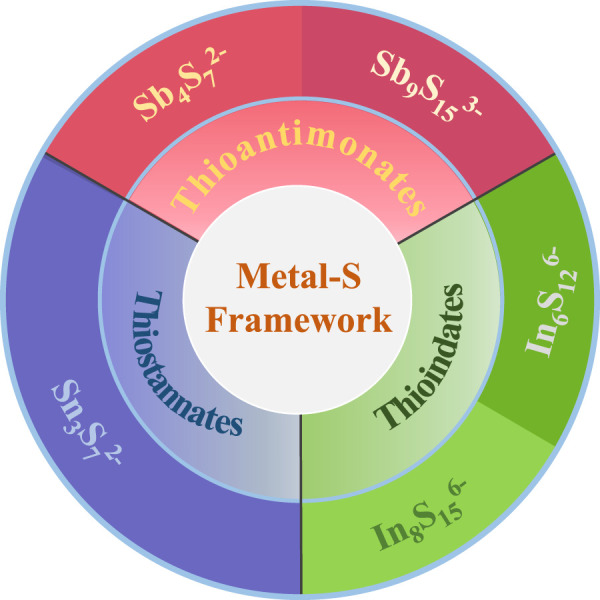
Categories of layered metal sulfides based on the framework composition (M_a_S_b_^c−^) reviewed in this paper.

Layered metal sulfides comprise the M_a_S_b_^c−^ framework and interlayer cations (Manos et al., 2008), where M represents the framework metals combined with S^2−^ ligands. These layered metal sulfides can be categorized into three groups based on their reported framework compositions: thioantimonates (Sb–S frameworks), thioindates (In–S frameworks), and thiostannates (Sn–S frameworks) (Figure 1). The charge-balancing cation in the interlayer can be adjusted according to the synthesis conditions (Manos and Kanatzidis, 2016). Various cations, such as K^+^, Na^+^, alkylammonium cations, and even complex organic cations in ionic liquids, have been intercalated into layered metal sulfides. The choice of exchangeable interlayer cations significantly influences the performance of ion exchangers (Kim et al., 2019). In this section, we outline the structural characteristics and Cs^+^ and Sr^2+^ removal efficiencies of layered metal sulfides with different metal–sulfur frameworks and interlayer cations.

### 3.1 Thioantimonate

Thioantimonates represent a recently developed class of layered metal sulfides. In the Sb–S frameworks of thioantimonates, Sb carries a positive trivalent charge and tends to adopt trigonal pyramidal coordination (Manos and Kanatzidis, 2016). Two prominent examples of thioantimonates are Sb_4_S_7_^2−^ and Sb_9_S_15_^3−^.

#### 3.1.1 Sb_4_S_7_^2−^

(NH_4_)_2_Sb_4_S_7_·2H_2_O, referred to as SbS-1, is a novel thioantimonate. In a recent study (Zhao et al., 2022b), single-crystal X-ray diffraction (XRD) analysis revealed that the crystal structure of SbS-1 was orthorhombic, with the space group *Pbca*. The asymmetric unit of SbS-1 consists of four Sb^3+^ ions, seven S^2−^ ions, and two NH_4_^+^ ions. The Sb^3+^ ions exhibit slightly distorted trigonal pyramidal coordination geometries. The formed {SbS_3_} units connect via corner-sharing S atoms to create a trimetallic pseudo-semicube cluster, {Sb_3_S_6_}. The {Sb_3_S_6_} cluster is further connected to two {SbS_3_} units by corner-sharing S atoms, resulting in an [Sb_4_S_7_]_*n*_^2*n*−^ chain along the [100] direction. Weak secondary interactions merge neighboring ribbons, leading to a double-chain structure. Within the crystal lattice, the *in situ* formed ammonium cations act as templates and counter ions, establishing extensive N-H···S hydrogen bonds, with the inorganic moieties surrounding the double ribbons.

**FIGURE 2:**
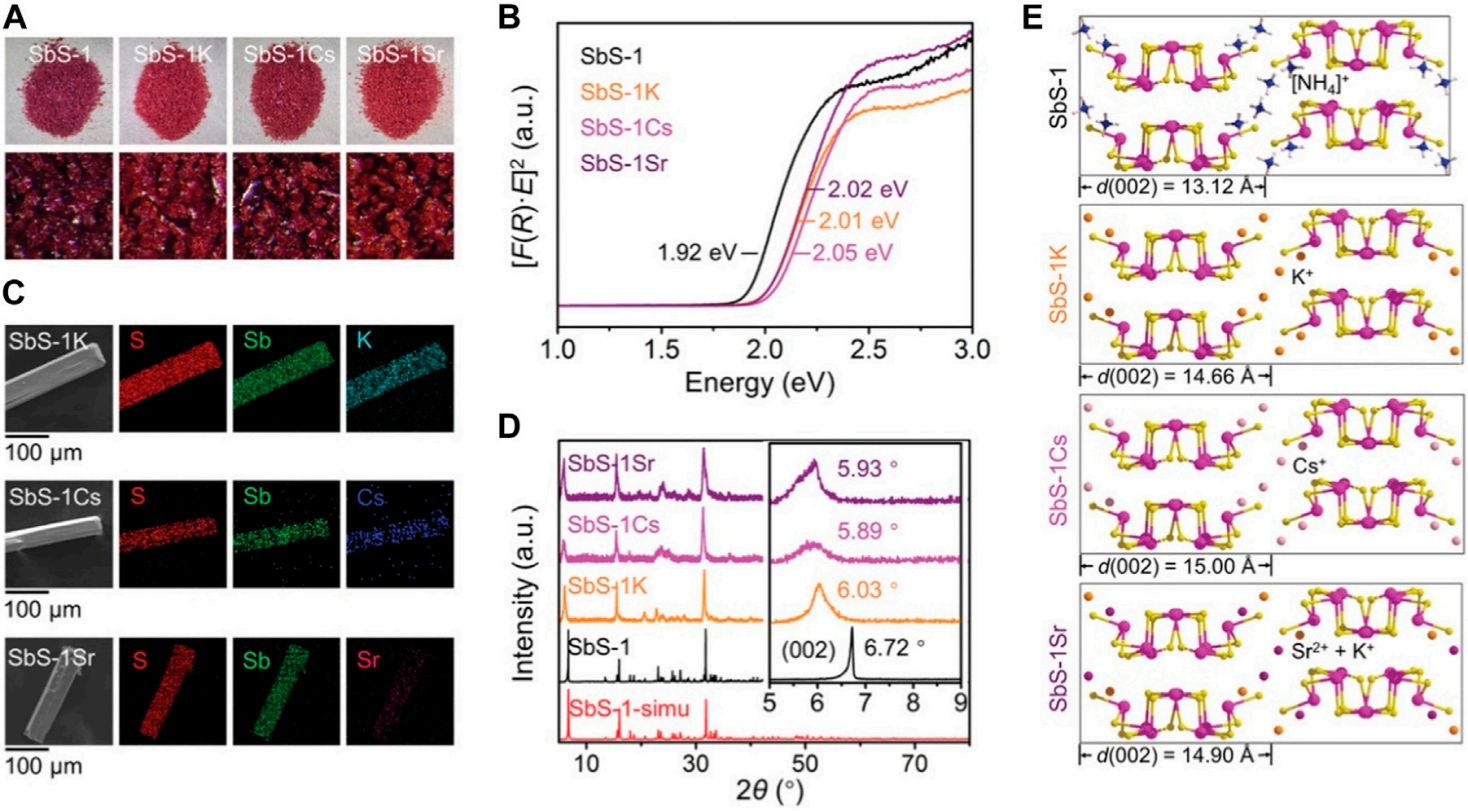
**(A)** Appearances, **(B)** Kubelka–Munk spectra, **(C)** microscopic morphologies and elemental mapping images, **(D)** powder XRD patterns, and **(E)** d(002)-spacing variation of pristine SbS-1, activated SbS-1K, and ion-exchanged products SbS-1Cs and SbS-1Sr. Reprinted with permission from Zhao et al. (2022b). Copyright from John Wiley and Sons (2022).

In Zhao et al. (2022b), SbS-1 exhibited relatively low exchange performance for Cs^+^ and Sr^2+^, probably owing to the strong hydrogen bonding interactions between NH_4_^+^ and [Sb_4_S_7_]_*n*_^2*n*−^ ribbons. To enhance the exchange capabilities, SbS-1 was transformed into K_2_Sb_4_S_7_·2H_2_O (referred to as SbS-1K) through washing with a KCl solution. The introduction of harder hydrated K^+^ significantly improved the exchange performance of the material for both Cs^+^ and Sr^2+^, attributable to the expansion of the interlayer space facilitated by the effect of hydrated K^+^. Consistent crystal morphologies occurred in the activated product SbS-1K and the Cs/Sr-exchanged products, abbreviated as SbS-1Cs and SbS-1Sr, respectively (Figure 2A). Notably, these products featured none of the distinct rough surfaces caused by fractures. The optical band featured an apparent blue shift, as observed in the Kubelka-Munk spectra (Figure 2B), after K^+^ activation and subsequent ion exchange with Cs^+^ and Sr^2+^. This phenomenon explains the slight color change to a brighter red (Figure 2A) (Sivakumar and Manikandan, 2019). Scanning electron microscopy (SEM) revealed the microscopic morphologies of SbS-K, SbS-Cs, and SbS-Sr, while the even distribution of K, Cs, and Sr throughout the samples was evident from elemental mapping images (Figure 2C). The results of elemental and thermogravimetric analysis indicated that K^+^ could be entirely replaced by Cs^+^ and partially replaced by Sr^2+^. The possible ion exchange reactions can be expressed by the following equations:

$$K_{2}{\text{Sb}}_{4}S_{7}∙2H_{2}O+2{\text{Cs}}^{+}\to {\text{Cs}}_{2}{\text{Sb}}_{4}S_{7}∙2H_{2}O+2K^{+}$$ (1)

$$K_{2}{\text{Sb}}_{4}S_{7}∙2H_{2}O+0.5{\text{Sr}}^{2+}\to {\text{Sr}}_{0.5}{\text{KSb}}_{4}S_{7}∙2H_{2}O+K^{+}$$ (2)

Powder XRD analysis was conducted to investigate changes in crystal structures before and after ion exchange. SbS-1K, SbS-1Cs, and SbS-1Sr exhibited the same crystal structure as the original SbS-1 (Figure 2D), indicating an isotactic ion-exchange process. The (002) Bragg peak in the XRD pattern shifted to lower 2*θ* values after ion exchange, indicating an increase in the interchain d(002)-spacing: from 13.12 Å for SbS-1 to 14.66 Å for SbS-1K, 15.00 Å for SbS-1Cs, and 14.90 Å for SbS-1Sr (Figure 2E). This spacing expansion is attributable to the insertion of larger cations, such as K^+^ (3.31 Å), Cs^+^ (3.29 Å), and Sr^2+^ (4.12 Å). Despite Sr^2+^ having a higher degree of hydration than Cs^+^, the non-equimolar exchange of K^+^ by Sr^2+^ led to a slightly smaller d(002)-spacing for SbS-1Sr than for SbS-1Cs, as shown in Eq. 2. An overall affinity sequence for the SbS-1K exchanger is Cs^+^ > K^+^ > H^+^ > Sr^2+^. The saturated Cs^+^ and Sr^2+^ could be entirely eluted using a 2 mol/dm^3^ KCl solution for 24 h, as confirmed by the results of energy-dispersive X-ray spectroscopy (EDS) and elemental mapping analysis. The elution mechanism likely results from the competitive effect of high-concentration K^+^ with Cs^+^ or Sr^2+^. Moreover, SbS-1K exhibited structural stability across a wide pH range, from alkaline (pH 11) to highly acidic (3 mol/dm^3^ HCl) conditions, establishing it as one of the most stable reported ion exchangers. Notably, exposure to high-energy β and γ radiation, even up to 200 kGy and 100 kGy, respectively, did not cause any structural or crystalline damage to SbS-1K.

**FIGURE 3:**
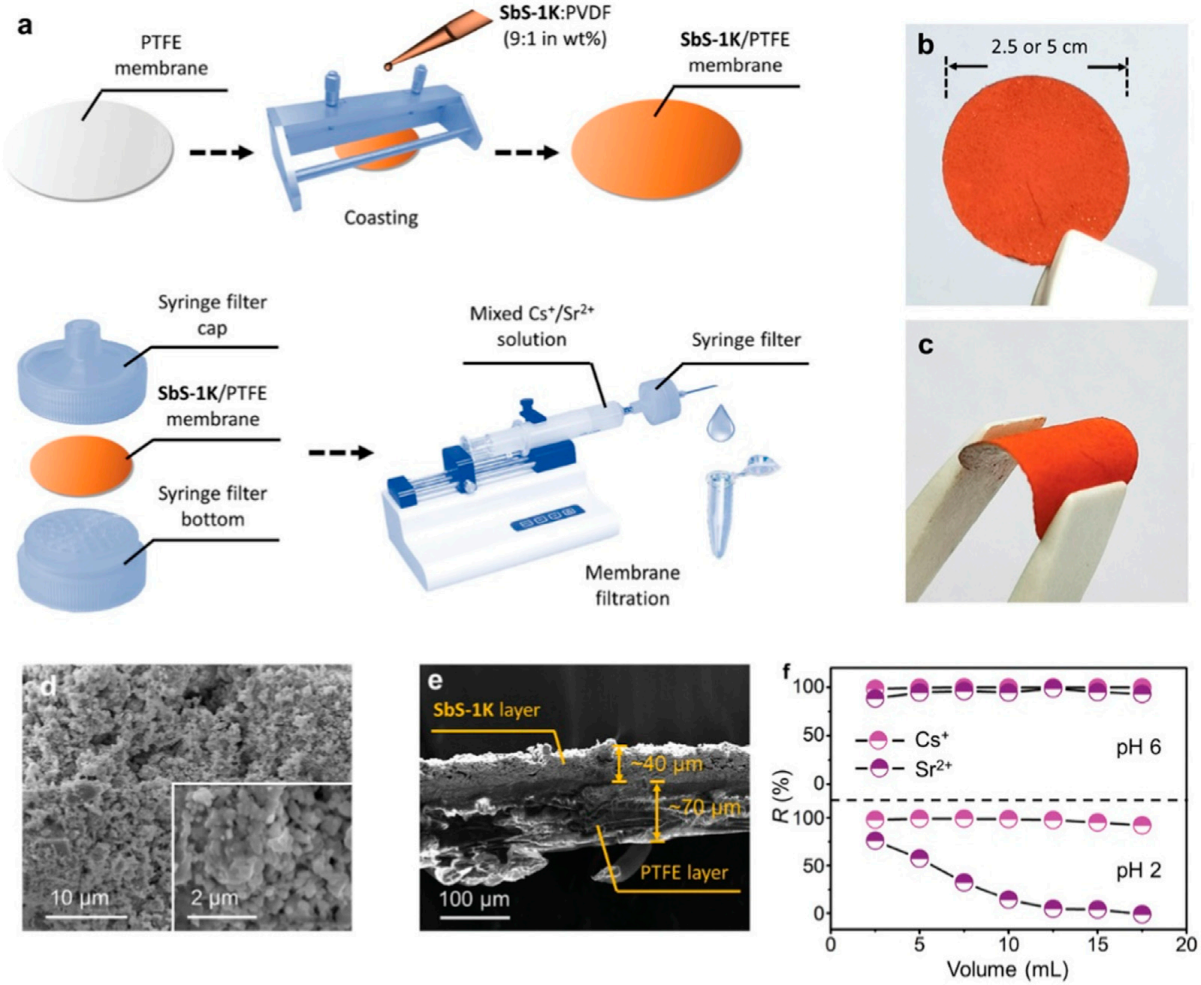
**(A)** Schematic of the membrane fabrication process and the membrane filtration operation. **(B,C)** External appearances of the SbS-1K/PTFE membrane in its flat and bent forms. **(D,E)** SEM image of the amplified surface and cross section of the SbS-1K/PTFE membrane. **(F)** Variation in Cs^+^ and Sr^2+^ removal efficiencies with effluent volume in the membrane filtration for a Cs^+^–Sr^2+^ mixed solution (*C*_0_ = 1 ppm for each) at pH 6 and pH 2. Reprinted with permission from Zhao et al. (2022b). Copyright from John Wiley and Sons (2022).

To further explore its application, SbS-1K was incorporated into a membrane for Cs^+^ and Sr^2+^. The ball-ground SbS-1K powder was mixed with polyvinylidene difluoride in a 9:1 weight ratio and cast onto a microporous polytetrafluoroethylene (PTFE) substrate. The resulting material was dried under a vacuum. The process for preparing SbS-1K/PTFE membrane is illustrated in Figure 3A. The fabricated SbS-1K/PTFE membrane was encapsulated between a pair of syringe filter tops and bottoms for subsequent injector-driven filtration. The paper-like SbS-1K/PTFE membrane had an orange color, smooth surface, and high flexibility (Figures 3B, C). Microscopic morphologies of the membrane surface, obtained via a low-magnification SEM, revealed numerous crystal particles with diameters of 0.1–0.3 µm, forming an interpenetrating porous network in the SbS-1K layer (Figure 3D). The SbS-1K layer and PTFE substrate were 40 and 70 µm thick, respectively (Figure 3E). The Cs^+^ and Sr^2+^ removal efficiencies of the SbS-1K/PTFE membrane during continuous filtration remained consistently high (>85%) at pH 6 (Figure 3F **)**. However, the Sr^2+^ removal efficiency decreased to 5% at pH 2 with increasing effluent volume. This pH-dependent functional switch demonstrates the potential of the SbS-1K/PTFE membrane for the effective co-exchange and separation of Cs^+^ and Sr^2+^.

#### 3.1.2 Sb_9_S_15_^3−^

(MeNH_3_)_3_Sb_9_S_15_, commonly referred to as FJSM-SbS, is another representative example of thioantimonates (Liao et al., 2021). Liao et al. (2021) reported that FJSM-SbS occurred as brownish-red flake-like crystals and crystallized in the orthorhombic space group *Cmc*2_1_. The asymmetric unit consists of 10 unique Sb sites, 16 S sites, and 4 MeNH_3_^+^ sites. Three neighboring S atoms coordinate with the Sb atoms to form a trigonal pyramid {SbS_3_} with Sb–S bond lengths and S–Sb–S angles similar to those observed in some reported thioantimonates (Zhao et al., 2022b). These {SbS_3_} units are interconnected through corner-sharing, resulting in a trinuclear cluster {Sb_3_S_6_}, while other{SbS_3_} units are linked via corner-sharing to form a linear trinuclear unit {Sb_3_S_7_}. The alternating arrangements of {Sb_3_S_6_} and {Sb_3_S_7_} clusters result in the formation of a 10-membered ring {Sb_10_S_10_}. These rings are interlinked through shared {Sb_3_S_7_} units, giving rise to an [Sb_9_S_15_]_*n*_^3*n−*^ double chain along the *b*-axis.

The structural changes during Cs^+^ exchange and subsequent elution by K^+^ were confirmed through a comparison of experimental XRD patterns with corresponding simulated patterns derived from single-crystal structures. FJSM-SbS exhibited remarkable selectivity for Cs^+^ removal from tap and lake water. The exchanged Cs^+^ could be easily eluted through washing with an excess of KCl solution. The mapping and EDS results of eluted products suggest that Cs^+^ ions could be entirely replaced by K^+^ ions, and K^+^ ions show the homogeneous distribution in the eluted material FJSM-SbS. The resulting regenerated FJSM-SbS maintained a well-layered structure. FJSM-SbS crystals retained stable frameworks even in strongly acidic (pH 1.0) and alkaline solutions (pH 12.0), demonstrating their exceptional stability under strong acidic and alkaline conditions. Furthermore, the parent structure of FJSM-SbS remained intact even after exposure to up to 200 kGy of β or γ radiation, without experiencing any structural collapse.

Although thioantimonates exhibit good ion exchange performance for Cs^+^ and Sr^2+^ as mentioned above, it is important to note that the thioantimonate materials are probably acutely toxic when orally or inhalationally exposed, and they also pose risks to aquatic life with long-lasting effects (National center for biotechnology information, 2023). Therefore, the toxicity of thioantimonates must be carefully considered when used for effluent treatment.

### 3.2 Thioindate

Thioindates have rarely been reported as ion exchangers for radionuclide removal. Thioindates, consisting of In^3+^ and S^2−^, typically adopt tetrahedral coordination in their In–S frameworks (Manos and Kanatzidis, 2016). In the following section, we describe two thioindate materials, In_6_S_12_^6−^ and In_8_S_15_^6−^, and their applications in the removal of Sr^2+^ from solution.

#### 3.2.1 In_6_S_12_^6−^

**FIGURE 4:**
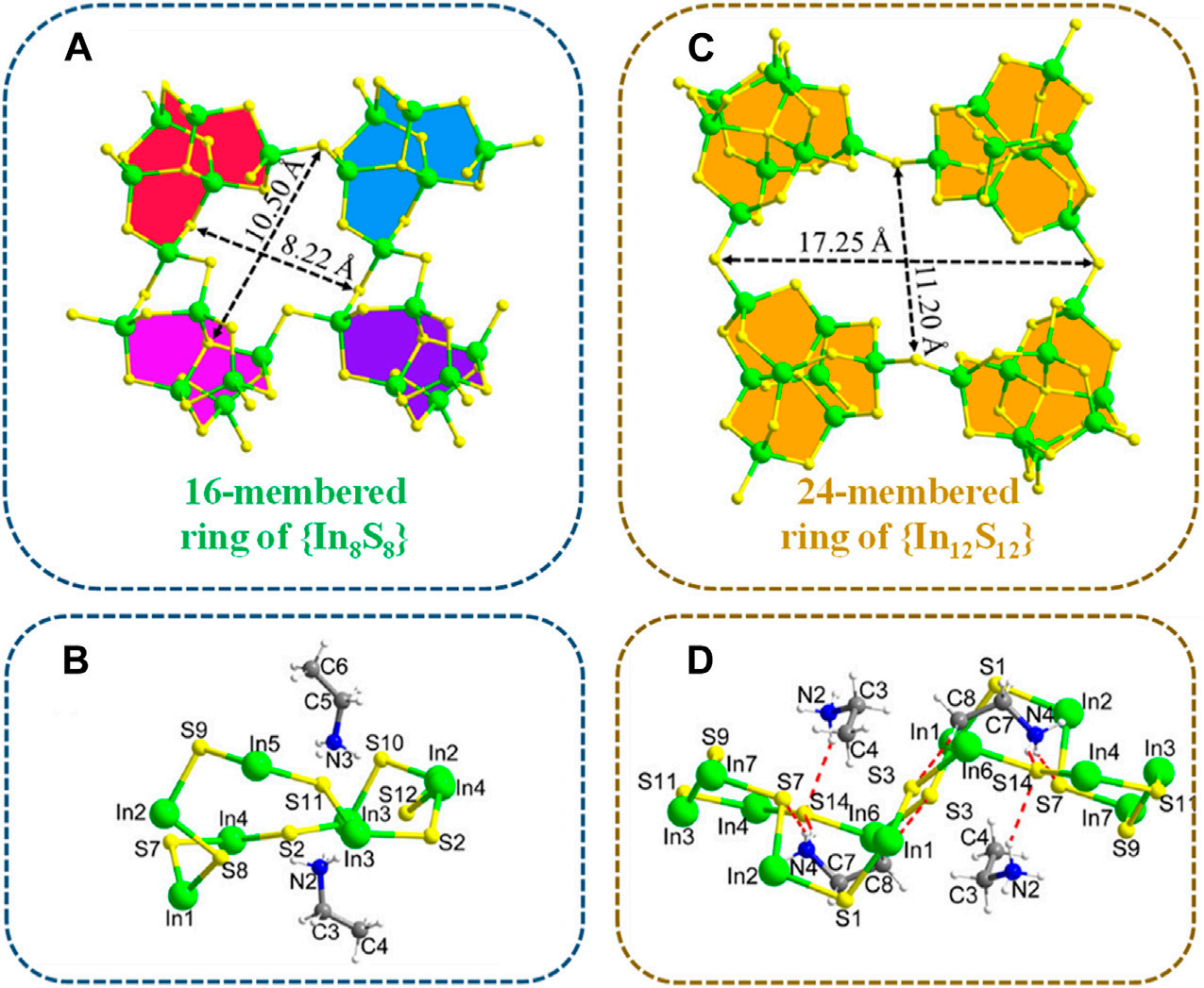
**(A)** 16-membered ring of {In_8_S_8_} and **(C)** 24-membered ring of {In_12_S_12_}. The template-ring relationship for the formation of **(B)** InS-1 and **(D)** InS-2. Adapted with permission from Sun et al. (2020). Copyright from American Chemical Society (2020).

Wang et al. (2020) discovered a novel ethylammonium-templated thioindate, (CH_3_CH_2_NH_3_)_6_In_6_S_12_ (referred to as InS-1). InS-1 crystallizes in the monoclinic *P*2_1_/*n* space group and is comprised of [In_6_S_12_]_*n*_^6*n*−^ anionic layers stacked with ethylammonium cations. The layer structure of InS-1 is built from the di-lacunary [In_6_S_15_]^12−^ cluster, which originates from the plenary P1-type [In_8_S_17_]^10−^ cluster through the removal of two [InS]^+^ groups. This partial vacancy leads to a reduced cluster size and an increased number of terminal S^2−^ ligands, resulting in a modified interlinkage mode. Within the structure, an {In_8_S_8_} ring is formed by one {In_3_S_4_} edge and three shorter edges, encompassing four [In_6_S_15_]^12−^ clusters (Figure 4A). The dimensions of the {In_8_S_8_} ring measure 10.50 × 8.22 Å^2^. Apart from two ethylammonium cations located above and below the ring plane, with their -NH_3_ groups oriented inwards into the cavity, no other entities are accommodated within the smaller {In_8_S_8_} ring in InS-1 (Figure 4B).

The exchange of Sr^2+^ with InS-1 was minimally affected by high concentrations of Na^+^, Mg^2+^, and Ca^2+^ but significantly influenced by K^+^. Consequently, the Sr^2+^-loaded product, InS-1Sr, could be efficiently regenerated using an excess of aqueous KCl solution via stirring. The eluted product InS-1K exhibited a complete replacement of Sr^2+^ by K^+^, as confirmed through EDS analysis, and showed a highly uniform distribution, as determined through elemental mapping. While InS-1K retained its crystal morphology after regeneration, its crystalline quality was slightly degraded owing to stacking deviations of the anionic layers caused by iterative cation exchange. Furthermore, the powder XRD patterns of InS-1 showed no signs of structural or crystalline degradation even when subjected to 200 kGy β or 100 kGy γ irradiation.

#### 3.2.2 In_8_S_15_^6−^

Another family of thioindates is based on In_3_S_12_^6−^ (Sun et al., 2020). A member of this family is (CH_3_CH_2_NH_3_)_6_In_8_S_15_, referred to as InS-2. The pale-yellow InS-2 crystallizes in the *P*2_1_/*n* space group and features densely packed [In_8_S_15_]_*n*_^6*n*−^ layers, with ethylammonium cations interspersed between the layers. P1-[In_8_S_17_]^10−^ clusters within the layer are interlinked by sharing terminal S atoms with four adjacent clusters. The ethylammonium cations reside in window cavities and interlamellar spaces, forming extensive hydrogen bonds with S atoms in the layers. The symmetrical integration of four P1-[In_8_S_17_]^10−^ clusters through corner-sharing results in the formation of a large and rhombic 24-membered ring denoted as {In_12_S_12_}, with diagonal dimensions measuring 17.25 × 11.20 Å^2^ (Figure 4C). The four {In_3_S_4_} edges of the {In_12_S_12_} ring, characterized by two terminal S atoms for each edge, are structurally identical, and each edge is exclusively derived from two terminal and one face {InS_4_} tetrahedra of each P1 cluster. The window of the {In_12_S_12_} ring within InS-2 appears larger than that of the 16-membered {In_8_S_8_} ring observed in the previously reported InS-1 compound (Wang et al., 2020). The formation of the {In_12_S_12_} ring in InS-2 is significantly influenced by the space-filling and shape-guiding role of ethylammonium. From a side view, the {In_12_S_12_} ring displays a triple-fold configuration, with each of the two-fold edges being occupied by one ethylammonium entity. The nonlinear C–C–N skeleton of the ethylammonium molecule appropriately fits within the concavity of the {In_12_S_12_} ring (Figure 4D).

The influence of the coexistence of Na^+^, K^+^, Mg^2+^, and Ca^2+^ on the ion exchange process of Sr^2+^ with InS-2 was investigated. Compared with Na^+^ and K^+^, Mg^2+^ and Ca^2+^ had a more pronounced negative impact on the exchange of Sr^2+^. This variation arises from the fact that Sr^2+^, Ca^2+^, and Mg^2+^ belong to the same group, sharing similar hydrated and structural ionic radii, 4.12 and 1.13 Å for Sr^2+^, 4.12 and 0.99 Å for Ca^2+^, and 4.28 and 0.65 Å for Mg^2+^, respectively (Nightingale, 1959). This similarity in ionic radii within the group leads to increased competition and interference during the ion exchange process, resulting in a more pronounced negative impact on the exchange of Sr^2+^. Furthermore, after Sr^2+^ exchange, there was an increase in pH under acidic conditions and a decrease in pH under alkaline conditions, indicating that InS-2 acted as a buffer to neutralize the solution. The exchanged Sr^2+^ could be efficiently eluted using a 2 mol/dm^3^ KCl solution. EDS analysis and elemental mapping confirmed the complete replacement of Sr^2+^ by K^+^ in the eluted product InS-2. XRD patterns of InS-2 indicated that the framework was retained after ion exchange, showcasing a high tolerance for cation transfer along the tunnels of InS-2. In contrast, the regeneration of InS-1 resulted in the destruction of its crystal structure. Moreover, InS-2 demonstrated remarkable structural stability and selective removal of Sr^2+^ even under strongly alkaline conditions (pH 14), outperforming most ion exchangers. However, XRD analysis revealed structural damage to InS-2 at pH 3, with complete decomposition occurring at pH < 2.

### 3.3 Thiostannate

Thiostannates have been extensively studied as a class of layered metal sulfides and have proven to be one of the most effective ion exchangers. The earliest report on thiostannates dates back to 1998 (Marking et al., 1998). The Sn_3_S_7_^2−^ framework, composed of Sn^4+^ and S^2−^, is a well-known example of thiostannates that exhibit various coordination geometries for the Sn^4+^, including tetrahedral, octahedral, or trigonal bipyramidal coordination (Manos and Kanatzidis, 2016).

#### 3.3.1 Sn_3_S_7_^2−^

**FIGURE 5:**
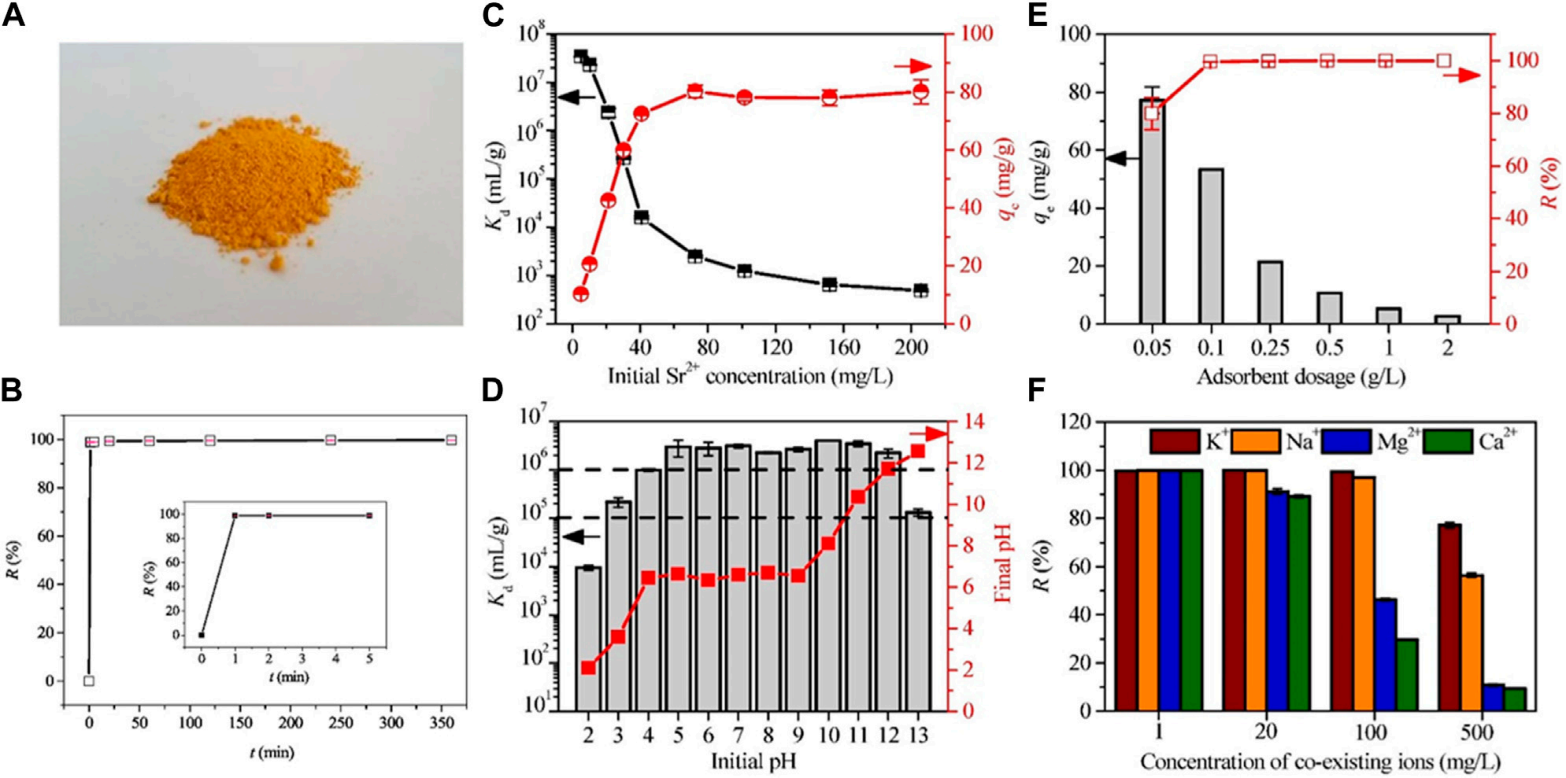
**(A)** Representative appearance of NaTS. Effects of **(B)** contact time (*C*_Sr_ = 5.0 mg/dm^3^, pH = 5.30, m/V = 0.5 g/dm^3^, *t* = 1–360 min), **(C)** initial Sr^2+^ concentration (pH = 5.5, m/V = 0.5 g/dm^3^, *t* = 2 h), **(D)** initial pH value (*C*_Sr_ = 5 mg/dm^3^, m/V = 0.5 g/dm^3^, *t* = 2 h), **(E)** adsorbent dosage (*C*_Sr_ = 5.0 mg/dm^3^, pH = 5.38, t = 2 h), and **(F)** the coexistence of Na^+^, K^+^, Ca^2+^, and Mg^2+^ (*C*_Sr_ = 5 mg/dm^3^, m/V = 0.5 g/dm^3^, *t* = 2 h) on the removal performance of Sr^2+^ by NaTS. Adapted with permission from Zhang et al. (2019b). Copyright from Elsevier (2019).

A rare example of a Na^+^-templated thiostannate based on the Sn_3_S_7_^2−^ framework was successfully synthesized by Zhang et al. through the hydrothermal method (Figure 5A). The synthesized material was identified as Na_2_Sn_3_S_7_ (referred to as NaTS) through semiquantitative EDS and X-ray photoelectron spectroscopy (Zhang et al., 2019b). However, the crystal structure of NaTS was not investigated in Zhang’s study. Figure 5B demonstrates the rapid kinetics of Sr^2+^ removal using NaTS. A high removal efficiency of over 98% was achieved within just 1 min. NaTS exhibited a strong affinity for Sr^2+^, particularly at low concentrations (Figure 5C). The distribution coefficient (*K*_d_, mL/g) is a crucial parameter to quantitatively describe the partitioning of the targeted metals between the solid and liquid phases. It can be calculated using the following equation:

$$K_{d}=\frac{C_{0}-C_{e}}{C_{e}}\times \frac{V}{m}$$ (3)

where *C*_0_ and *C*_e_ are the initial and equilibrium concentrations of the metal ions in the solution, *V* denotes the volume (mL) of the test solution, and *m* represents the mass (g) of the ion exchanger used in the experiment. The *K*_d_ values of NaTS reached as high as 10^6^ mL/g over a pH range of 4–12 (Figure 5D), owing to the robustness of the NaTS framework and its resilience to both acidic and alkaline conditions. However, the *K*_d_ values for Sr^2+^ decreased at pH 2 owing to the decomposition of the adsorbent under highly acidic conditions. The introduction of NaTS led to an increase in the initial pH under acidic conditions, but a reduction in the initial pH under alkaline conditions, indicating that NaTS could function as both a proton acceptor and donor, resulting in solution neutralization. The removal yield for Sr^2+^ nearly reached 100% when the absorbent dosage exceeded 0.1 g/dm^3^ (Figure 5E). Additionally, Sr^2+^ uptake by NaTS remained unaffected by the presence of Na^+^ and K^+^ (Zhang et al., 2016; Hong et al., 2017). However, the exchange of Sr^2+^ was significantly impeded by higher Ca^2+^ and Mg^2+^ concentrations (Figure 5F) (Tansel et al., 2006), likely owing to the difference in hydrated radii.

**FIGURE 6:**
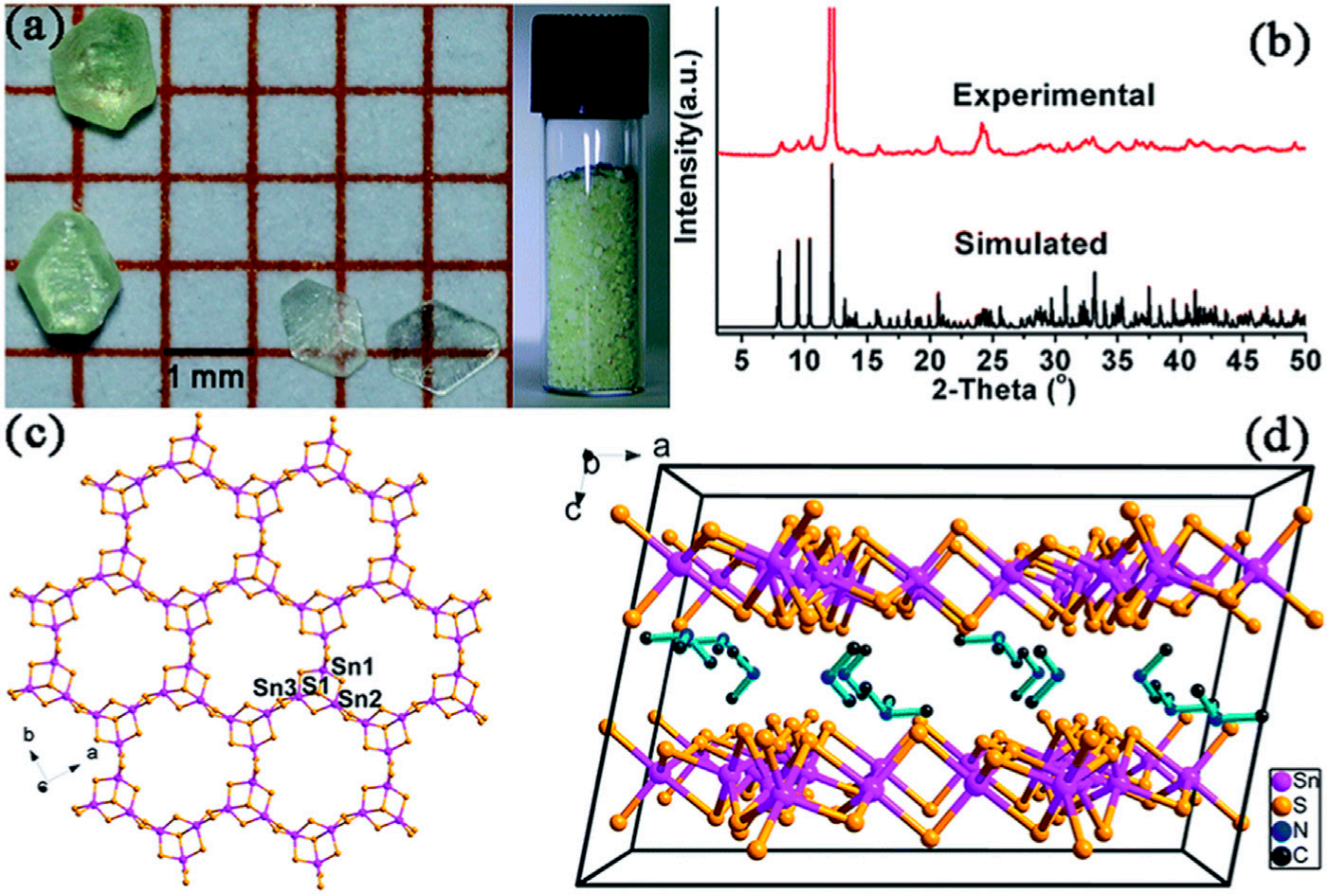
**(A)** Photographs showing FJSM-SnS crystals; **(B)** experimental and simulated powder XRD patterns; **(C)** a 2D [Sn_3_S_7_]_*n*_^2*n*−^ anionic layer oriented parallel to the *ab* plane; **(D)** packing arrangement of the layers along the *b*-axis. The H_2_O molecules and H atoms of organic amines are omitted for clarity. Reprinted with permission from Qi et al. (2015). Copyright from Royal Society of Chemistry (2015).

Qi et al. reported a stable yellow hexagonal thiostannate known as FJSM-SnS (Qi et al., 2015). This compound was synthesized through a solvothermal method and featured Sn_3_S_7_^2−^ with mixed templated MeNH_2_^+^ and Me_3_NH^+^ cations. The chemical formula of FJSM-SnS was determined as (Me_2_NH_2_)_4/3_(Me_3_NH)_2/3_(Sn_3_S_7_)·1.25H_2_O, consistent with the simulated XRD spectra (Figures 6A, B). Through single-crystal XRD, the crystal structure was found to belong to the *C*2/*c* space group. The Sn atoms in FJSM-SnS are five-coordinated with S, forming SnS_5_ trigonal bipyramids. These bipyramids are fused via edge-sharing to assemble an Sn_3_S_10_ unit with an Sn_3_S_4_ semi-cubane core. Three Sn_3_S_10_ units are connected through edge-sharing, resulting in a 2D [Sn_3_S_7_]_*n*_^2*n*−^ anionic layer. Within this layer, windows are formed by 24-membered Sn_12_S_12_ rings derived from six Sn_3_S_4_ cores (Figure 6C). The 2D [Sn_3_S_7_]_*n*_^2*n*−^ layers are stacked along the *c*-axis (Figure 6D). The interlayer spaces are occupied by highly disordered cations Me_2_NH_2_^+^ and Me_3_NH^+^ and lattice water molecules, with an estimated interlayer distance of 7.258 Å (Manos and Kanatzidis, 2012). The unexpected Me_3_NH^+^ cations are believed to be generated *in situ* from the solvent Me_2_NH, according to the results of mass spectra (Staelens et al., 2004).

FJSM-SnS was found to have excellent exchange properties for Cs^+^ and Sr^2+^. The maximum adsorption of these metal ions was achieved within 5 min at 65°C, and ion exchange equilibrium was reached within 30–60 min at room temperature. To test the performance of FJSM-SnS in simulated groundwater, FJSM-SnS was examined for Cs^+^ and Sr^2+^ removal in the presence of various coexisting cations such as Na^+^, K^+^, Ca^2+^, and Mg^2+^. The *K*_d_ for these ions generally decreased as follows: Sr^2+^ > Ca^2+^ > Mg^2+^ > Cs^2+^ > K^+^. Competitive ion exchange experiments using a 10:10:1 molar ratio of Na^+^, K^+^, and Cs^+^ and a 10:10:1 molar ratio of Mg^2+^, Ca^2+^, and Sr^2+^ showed that the *K*_d_ values of Cs^+^ or Sr^2+^ were still several times higher than those of alkali metals and alkaline earth metals, respectively. This suggests that FJSM-SnS was highly effective in removing Cs^+^ and Sr^2+^ even in the presence of large numbers of coexisting Na^+^, K^+^, Mg^2+^, and Ca^2+^ ions. The removal rate was higher at a lower V-to-m ratio (volume of the solution to the mass of the ion exchanger), indicating the availability of more exchange sites for enhancing removal efficiencies (Manos and Kanatzidis, 2009). Furthermore, FJSM-SnS could be used as a suitable stationary phase in an ion exchange column, with removal capacities of 96%–100% for Cs^+^ and Sr^2+^ even after passing 900-bed volumes through the ion exchange column (total volume passed = 2.42 L, 1-bed volume = 2.79 mL). To the best of our knowledge, Manos and Kanatzidis’s (2009) study represents one of the first reports of the use of a layered metal sulfide in columns.

**FIGURE 7:**
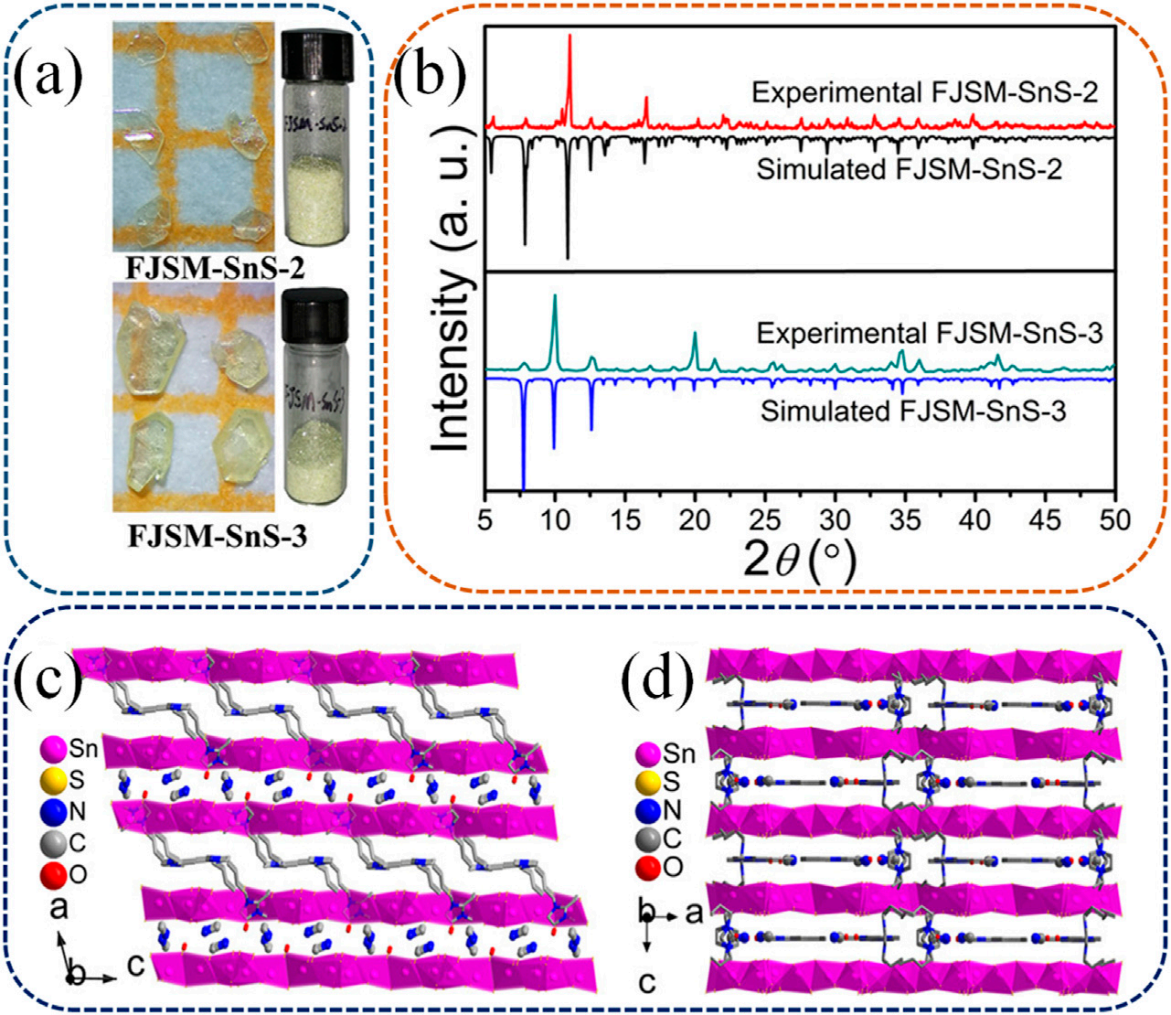
**(A)** Photographs of FJSM-SnS-2 and FJSM-SnS-3. **(B)** Experimental and simulated powder XRD patterns of FJSM-SnS-2 and FJSM-SnS-3. The arrangement of [CH_3_NH_3_]^+^ and [Bmmim]^+^ at the adjacent interlayer spaces of FJSM-SnS-2 **(C)** and FJSM-SnS-3 **(D)**. H atoms are omitted for clarity. Reprinted with permission from Li W. et al. (2021). Copyright from American Chemical Society (2021).

In a more recent study by Li et al., new members of the Sn_3_S_7_^2−^ family with templated mixed cations of CH_3_NH_3_^+^ and Bmmim^+^ (1-butyl-2,3-dimethylimidazolium) emerged as superior ion exchange materials (Li et al., 2021b). Two crystals were identified as [CH_3_NH_3_][Bmmim]Sn_3_S_7_·0.5H_2_O (referred to as FJSM-SnS-2) and (CH_3_NH_3_)_0.75_(Bmmim)_1.25_Sn_3_S_7_·H_2_O (referred to as FJSM-SnS-3). This is the first instance of simultaneously incorporating protonated organic amine and ionic liquid cations into layered metal sulfides to prepare an ion exchange material. The exchangeability of large-sized Bmmim^+^ with Cs^+^ and Sr^2+^ was demonstrated through mass spectrometry-based *in situ* tracking. Figures 7A, B depict the photographs, experimental data, and simulated XRD patterns of FJSM-SnS-2 and FJSM-SnS-3. FJSM-SnS-2 and FJSM-SnS-3 differed in the ratios and arrangements of the mixed cations within the interlayer spaces. In FJSM-SnS-2, CH_3_NH_3_^+^ and Bmmim^+^ were alternately arranged in different interlayer spaces (Figure 7C), while in FJSM-SnS-3, they occupied the same interlayer spaces (Figure 7D).

The Cs^+^ and Sr^2+^ exchange properties of both FJSM-SnS-2 and FJSM-SnS-3 were systematically studied. These compounds exhibited high capacities, rapid kinetics, a wide pH range for Cs^+^ and Sr^2+^ removal, and convenient elution. Notably, both compounds performed excellently in scavenging Sr^2+^, even in the presence of high concentrations of interfering Na^+^ and in contaminated tap and lake water. Although both compounds exhibited the same total amounts of exchangeable cations between the interlayer spaces, they displayed significant differences in Cs^+^ exchange capacities: FJSM-SnS-2 yielded a capacity twice that of FJSM-SnS-3. This discrepancy is attributable to the CH_3_NH_3_^+^ and Bmmim^+^ in FJSM-SnS-2 being located between interlayer spaces, providing a smoother pathway for Cs^+^ exchange. In contrast, the coexistence of exchangeable cations within the same interlayer space in FJSM-SnS-3 could impede each other’s escape from the structure, potentially limiting the ion exchange capacity. Conversely, both compounds exhibited similar Sr^2+^ exchange capacities, which appeared to be unaffected by the arrangement of the cations. This phenomenon is attributable to the fact that the high-valency Sr^2+^ has stronger interactions with the Lewis base and anionic layered sulfide network, which override the effects of cation arrangement. Furthermore, the Cs^+^ and Sr^2+^ exchange properties of FJSM-SnS-2 and FJSM-SnS-3 were investigated before and after exposure to β and γ radiation. Both compounds exhibited excellent structural stability and retained high removal efficiency even after intense β and γ radiation of up to 200 kGy.

**FIGURE 8:**
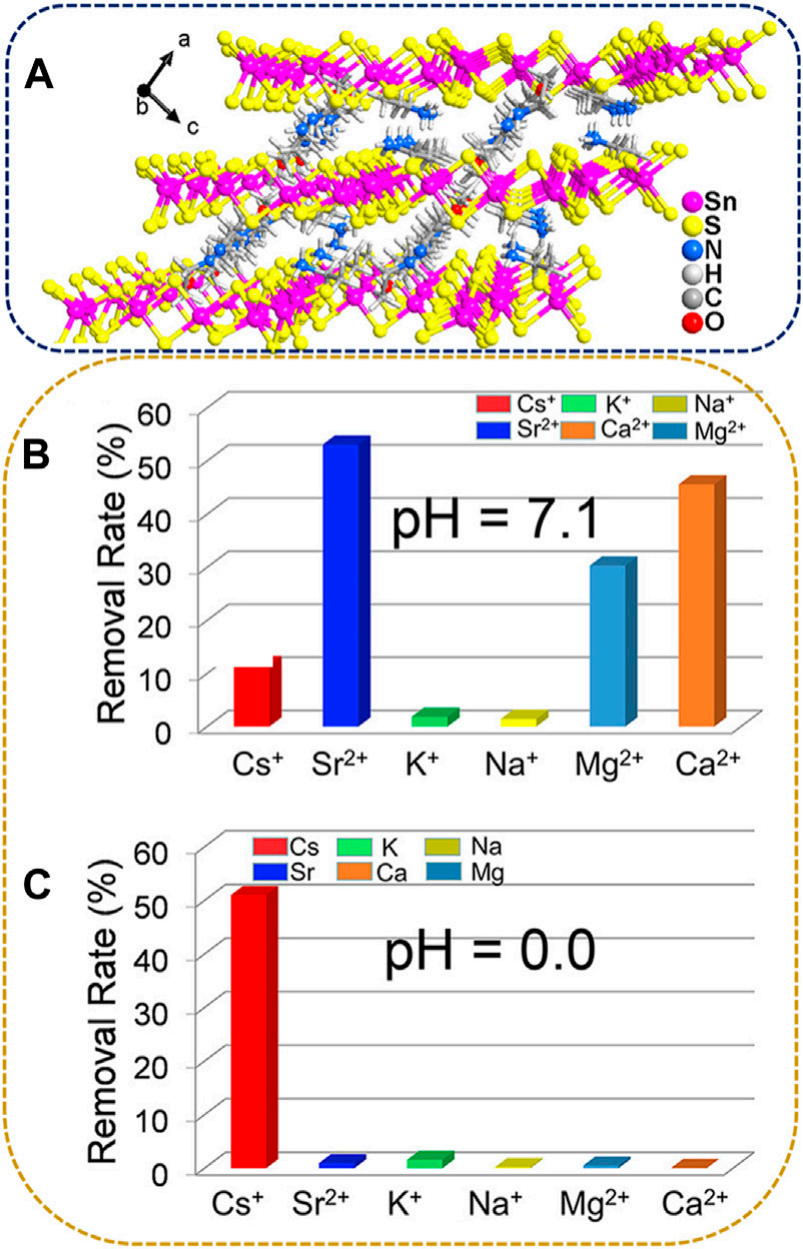
**(A)** Stacking of the [Sn_3_S_7_]_*n*_^2*n*−^ layers in FJSM-SnS-4 viewed along the *b*-axis. Cs^+^, Sr^2+^, K^+^, Na^+^, Mg^2+^, and Ca^2+^ removal efficiencies of FJSM-SnS-4 under **(B)** neutral or **(C)** acidic conditions. Here [Cs, Sr] = 5.72–6.43 mg/dm^3^ and [Na, K, Mg, Ca] = 44.15–51.71 mg/dm^3^. Adapted with permission from Li et al. (2021a). Copyright from American Chemical Society (2021).

Another example of thiostannates with the Sn_3_S_7_^2−^ layered structure involves the incorporation of organic amines as interlayer organic cations. (Li et al., 2021a). synthesized a compound identified as [(EtNH_3_)_1.68_(Et_2_NH_2_)_0.32_]Sn_3_S_7_·0.68H_2_O (referred to as FJSM-SnS-4) through X-ray refinement. FJSM-SnS-4 belongs to the *I*2/*a* space group, as revealed via XRD analysis. The crystal structure features a microporous anionic layer of [Sn_3_S_7_]_*n*_^2*n*−^ with protonated ethylamine and diethylamine cations serving as templates within the interlayer spaces. All Sn^4+^ ions are five-coordinated with S^2-^, forming {SnS_5_} trigonal bipyramids. These {SnS_5_} units further combine through edge-sharing to form a secondary building unit known as {Sn_3_S_10_}. The {Sn_3_S_10_} unit has a semicubic {Sn_3_S_4_} core. Six {Sn_3_S_4_} nodes assemble to form a hexagonal window comprising a 24-membered ring. Through edge-sharing, the hexagonal windows result in the formation of the 2D anionic layer of [Sn_3_S_7_]_*n*_^2*n*−^ parallel to the *b*-*c* plane. Protonated organic amine cations form N-H···S and C-H···S hydrogen bonds with S^2−^ from the adjacent layer, stabilizing the structure (Figure 8A).

Li et al. also investigated the selectivity of FJSM-SnS-4 for Cs^+^ and Sr^2+^ against Na^+^, K^+^, Ca^2+^, and Mg^2+^ in neutral (pH 7) and acidic (pH 0) solutions. The affinity of FJSM-SnS-4 toward the metal cations decreased as follows in a neutral environment: Sr^2+^ > Ca^2+^ > Mg^2+^ > Cs^+^ > K^+^ > Na^+^ (Figure 8B). Moreover, the presence of excess Ca^2+^ and Mg^2+^ significantly interfered with the Sr^2+^ exchange process. However, the Cs^+^ removal efficiency reached 51.01% under highly acidic conditions (pH = 0), whereas the removal efficiencies of the other ions, Na^+^, K^+^, Mg^2+^, Ca^2+^, and Sr^2+^, were very low (<5%, Figure 8C). This phenomenon indicates that FJSM-SnS-4 could effectively remove and selectively separate Cs^+^ and Sr^2+^ by controlling pH, even in the presence of various coexisting cations. In addition, the stable structure of FJSM-SnS-4 provided excellent resistance to acidity and β and γ irradiation.

**FIGURE 9:**
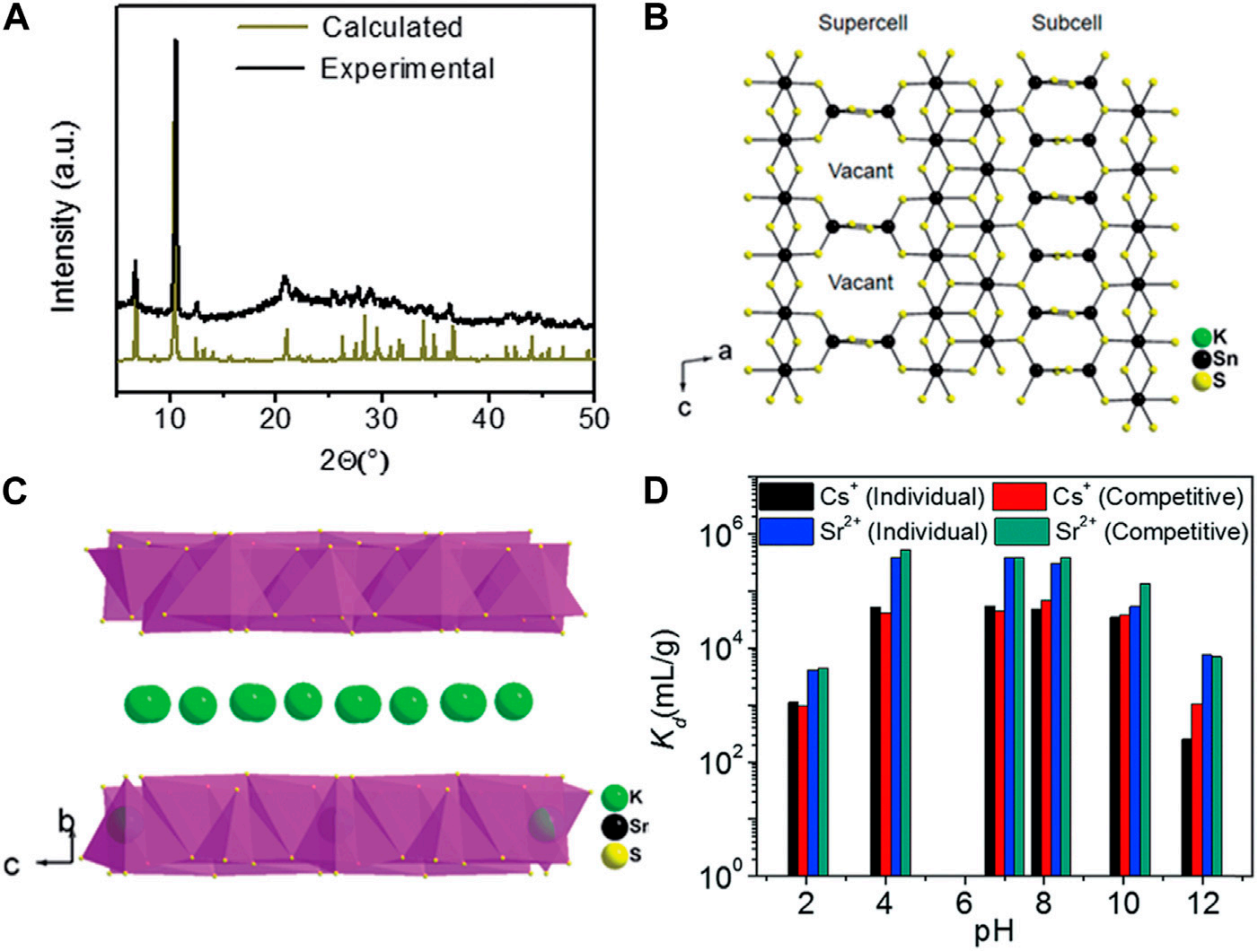
**(A)** Calculated and experimental powder XRD pattern of KTS-3. **(B)** [Sn_3_S_7_]^2−^ layer with ideally ordered Sn and vacancy sites. Refinement suggests the presence of some Sn atoms in the vacancies with a fractional occupancy of 31.1% owing to the diffuse nature of the supercell reflections. **(C)** Schematic of the layer structure and interlayer K^+^. **(D)** Variation in the *K*_d_ of individual and competitive Cs^+^ and Sr^2+^ exchange with pH. The initial concentrations of Cs^+^ and Sr^2+^ were 7.4 and 6.9 ppm, respectively, and the V/m ratio was 1 dm^3^/g. Adapted with permission from (Sarma et al., 2016). Copyright from Royal Society of Chemistry (2016).

K_2x_Sn_4-x_S_8-x_ (x = 0.65–1), abbreviated as KTS-3, is another thiostannate that exhibits excellent potential for ion exchange applications in the removal of Cs^+^ and Sr^2+^ (Sarma et al., 2016). Powder XRD analysis confirmed that the synthesized materials closely matched the calculated pattern obtained from the single-crystal model (Figure 9A). Further investigations using single-crystal XRD measurements revealed that the structure of KTS-3 is based on {SnS_6_} octahedra forming ribbons that extend along the *c*-axis. These ribbons are interconnected by {SnS_4_} units, which take the form of {Sn_2_S_6_} bridges (Figure 9B). The interlayer space within the structure is occupied by K^+^, which compensates for the negative charge of the anionic layers (Figure 9C). The K^+^ ions within the interlayer spaces exhibit high disorder and mobility, making them easily exchangeable with various other cations. Both the parent and exchanged KTS-3 are characterized by an isotactic structure. The authors proposed ion exchange processes for Cs^+^ and Sr^2+^, and the chemical equations are presented using K_1.92_Sn_3.04_S_7.04_ as an example.

$$K_{1.92}{\text{Sn}}_{3.04}S_{7.04}+1.92{\text{Cs}}^{+}\to {\text{Cs}}_{1.92}{\text{Sn}}_{3.04}S_{7.04}+1.92K^{+}$$ (4)

$$K_{1.92}{\text{Sn}}_{3.04}S_{7.04}+0.96{\text{Sr}}^{2+}+yH_{2}O\to [\text{Sr}{{\left(H_{2}O\right)}_{y}]\text{Sn}}_{3.04}S_{7.04}+{1.92K}^{+}$$ (5)

Ion exchange experiments with KTS-3 for individual and competitive removal of Cs^+^ and Sr^2+^ were conducted across a range of pH values (Figure 9D). Over 97% of Cs^+^ was effectively removed by KTS-3 at pH 4–10. The Cs^+^ removal efficiencies remained at ∼53% even in highly acidic environments with a pH of 2. The *K*_d_ values for Sr^2+^ were greater than 10^4^ at pH 4–10, significantly higher than those for Cs^+^. However, the *K*_d_ values for Cs^+^ and Sr^2+^ pH 2 and 12 were remarkably lower than those at pH 4–10, attributable to the partial decomposition of KTS-3 in strongly acidic and alkaline environments. Interestingly, KTS-3 also exhibited high selectivity for Cs^+^ in the presence of Na^+^ (0.1 mol/dm^3^), while the *K*_d_ values for Sr^2+^ significantly decreased with increasing Na^+^ concentration.

## 4 Comparison of the Cs^+^ and Sr^2+^ removal performances of ion exchangers

The maximum ion exchange capacity (*q*_m_) is a crucial parameter for evaluating the performance of ion exchangers. It can be determined using the Langmuir model, as described below (Guo and Wang, 2019):

$$q=q_{m}\frac{bC_{e}}{1+bC_{e}}\;$$ (6)

where *q* (mg/g) is the number of cations exchanged in the solid phase at equilibrium, *q*_m_ (mg/g) is the maximum ion-exchange capacity, *b* (dm^3^/mg) is the Langmuir constant related to the free energy of the ion exchange process, and *C*_e_ (ppm) is the equilibrium concentration in the liquid phase.

**FIGURE 10:**
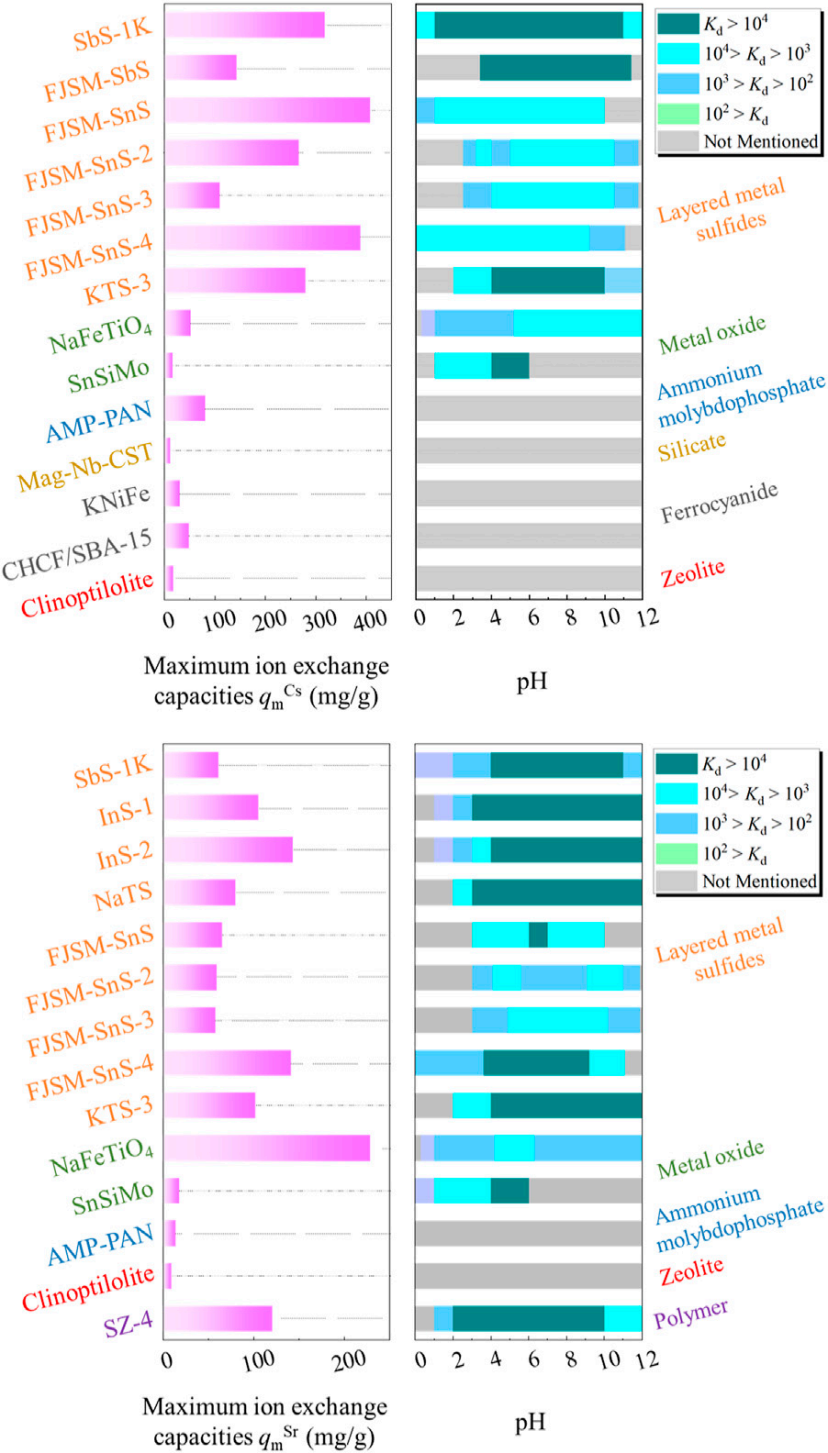
Maximum ion exchange capacities *q*_m_^Cs^ and *q*_m_^Sr^ achieved using layered metal sulfides and other typical ion exchange materials, and the dependence of *K*_d_ values on the pH active windows. The corresponding color-coded labels indicate the types of ion exchangers. The *q*_m_ values are estimated through data fitting with the Langmuir model. The data for NaFeTiO_4_, [(SnO_2_)_3_·(H_2_SiO_3_)·(H_2_MoO_4_)_3_]·6H_2_O (SnSiMo), (NH_4_)_3_[PMo_12_O_36_]·polyacrylonitrile (AMP-PAN), magnetic Nb-substituted crystalline silicotitanate (Mag-Nb-CST), K_1.34_Ni_0.33_[NiFe(CN)_6_] (KNiFe), SBA-15 embedded CuFe(CN)_6_ (CHCF/SBA-15), clinoptilolite, and [(CH)_3_NH_2_][ZrCH_2_(PO_3_)_2_F] (SZ-4) are obtained from Smičiklas et al. (2007), Park et al. (2010), Michel et al. (2015), Zhao et al. (2018), Abdel-Galil et al. (2019), Zhang et al. (2019a), Xiang et al. (2019), Amesh et al. (2020), respectively.

Figure 10 summarizes the *q*_m_ and *K*_d_ values of layered metal sulfides and compares their removal performances against other typical ion exchangers. For Cs^+^ removal, layered metal sulfides exhibited impressive *q*_m_^Cs^ values ranging from 109.6 to 408.9 mg/g, outperforming other types of adsorbents. Among them, FJSM-SnS exhibited the maximum *q*_m_, which is eight times that of clinoptilolite. However, the *q*_m_^Sr^ values for layered metal sulfides ranged from 57.8 to 143.3 mg/g, lagging behind those of NaFeTiO_4_ and SZ-4. Notably, the practical-to-theoretical capacity ratios for Sr^2+^ were markedly lower than those for Cs^+^ across all the layered metal sulfides. For instance, the *q*_m_^Cs^ and *q*_m_^Sr^ values for SbS-1K were determined to be 318.77 and 61.12 mg/g, respectively, representing 99.03% and 57.60% of the theoretical ion exchange capacities (321.90 mg/g for Cs^+^, 106.11 mg/g for Sr^2+^) of SbS-1K. This phenomenon is attributable to the non-equimolar exchange of interlayer ions with Sr^2+^ (Manos et al., 2008) and the insufficient interlamellar space available to accommodate the total stoichiometric number of large hydrated Sr^2+^ (e.g., [Sr(H_2_O)_6_]^2+^) (Sun et al., 2020).

The distribution coefficients of the ion exchanger, *K*_d_, are influenced by various factors, including the initial metal concentration, temperature, and specific surface area of materials. Therefore, this review presents the pH windows within which the observed *K*_d_ values fall, rather than directly comparing *K*_d_ values. This approach comprehensively delineates the ion exchange performance of layered metal sulfides. Compared with other materials, layered metal sulfides exhibited a wider pH range with higher *K*_d_ values for Cs^+^, demonstrating their superiority to other ion exchangers. SbS-1K exhibited the widest pH range (pH 1–11) for optimal Cs^+^ removal. This suggests that layered metal sulfides are well-suited for a range of pH conditions, making them versatile and practical ion exchangers for Cs^+^ removal. Regarding Sr^2+^ removal, the pH windows of *K*_d_ values highlight that layered metal sulfides are particularly suitable for strongly alkaline environments. These findings demonstrate the application potential of layered metal sulfides for Sr^2+^ removal in strongly alkaline environments, whereas other ion exchangers may not perform as effectively under the same conditions.

In the evaluation of the removal efficiency of radionuclides, considering the structural stability against irradiation is vital. The β and γ irradiation resulting from radionuclide decay can lead to the dissociation of H_2_O molecules and the generation of highly reactive radical products, including HO^·^, H^‧^, and H_2_O_2_. These radicals possess the potential to react with organic molecules and metal–oxygen bonds, thereby jeopardizing the integrity of material structures. However, the metal–sulfur bonds that characterize layered metal sulfides are more resistant to free radical attack than metal–oxygen bonds, probably owing to the lower energy associated with metal–sulfur bonding (Sarkar et al., 2022). Consequently, numerous reports have indicated that most layered metal sulfides exhibited excellent radiation resistance, maintaining their structural integrity and ion exchange stability even after exposure to high doses of β/γ radiation, reaching up to 200 kGy. In contrast, other organic ion exchangers, such as polymer SZ-4, might experience degradation under similar conditions, despite their satisfactory ion exchange performance (Hang et al., 2013; Kopal et al., 2018).

## 5 Conclusion and challenges

The selective removal of hazardous isotopes, such as ^137^Cs and ^90^Sr, from radioactive effluents is significant for both environmental conservation and human wellbeing. In recent years, layered metal sulfides have emerged as highly promising ion exchangers. This paper provides a comprehensive overview of the progress made in the use of layered metal sulfides for the removal of Cs^+^ and Sr^2+^ from aqueous environments. The layered metal sulfides featuring M_a_S_b_^c−^ frameworks are categorized into three distinct groups according to the various metals associated with S^2−^: thioantimonates, thioindates, and thiostannates. This review discusses their synthesis methods, crystal structures, and Cs^+^ and Sr^2+^ removal performances. Moreover, layered metal sulfides are compared with conventional ion exchangers, allowing for a comprehensive evaluation of the effectiveness of the sulfide compounds. Layered metal sulfides exhibit unique advantages for Cs^+^ and Sr^2+^ removal, including their high ion exchange capacities, wide active pH ranges with high *K*_d_ values, and remarkable resistance to irradiation. These attributes are superior to those of other ion exchange materials. Overall, this review comprehensively presents the advances in the utilization of layered metal sulfides to remove Cs^+^ and Sr^2+^ from aqueous environments, and it is a valuable resource for researchers and practitioners in the field.

In recent years, considerable efforts have been dedicated to exploring the practical application of layered metal sulfides as ion exchangers. However, several challenges still need to be addressed before the materials can be commercially implemented. Below, we present our perspective on some of these challenges, focusing on the development of layered metal sulfides and their practical application in the field of radionuclide removal.

1. Preparation method

The advancement of layered metal sulfides greatly depends on the enhancement of synthesis techniques. Presently, the synthesis of layered metal sulfides is conducted at a multigram scale through hydrothermal or solvothermal methods (Manos and Kanatzidis, 2016). However, challenges such as relatively high cost, equipment complexity, and time-intensive processes impede the practical viability of these methodologies for commercial purposes. Moreover, the crystals of layered metal sulfides obtained through hydrothermal or solvothermal methods often exhibit ultrafine morphologies and weak mechanical strength, thereby presenting obstacles for column operations involving these materials. Additionally, the variety of exchangeable cations templated within layered metal sulfides remains limited due to synthesis method constraints (Li et al., 2021b), mainly focusing on K^+^ and organic amines. To overcome these challenges, there is a significant need to explore low-cost and easy-to-operate synthesis methods that maintain high purity and ion exchange performance. Alternative strategies such as vacuum calcination or ball milling methods may offer solutions to these issues.

2. Competition effects of coexisting cations

In current ion exchange experiments, Cs^+^ and Sr^2+^ concentrations are often artificially elevated beyond levels encountered in practical scenarios, which benefits the accurate analysis of their concentrations through inductively coupled plasma techniques. However, this approach tends to underestimate the competitive effects of coexisting cations, such as Na^+^, K^+^, Ca^2+^, and Mg^2+^. While numerous ion exchangers have exhibited high efficiency in simulated solutions, their real-world performance in effluents is often compromised by significant interference from competing ions. Therefore, further research should focus on conducting ion exchange experiments using trace concentrations of Cs^+^ and Sr^2+^ to better elucidate and evaluate the effectiveness of layered metal sulfides in the presence of realistic coexisting cation concentrations.

3. Treatment of spent layered metal sulfides

Proper treatment of spent layered metal sulfides is a crucial procedure that needs to be considered in the application of these materials to radionuclide removal. Although some studies have investigated the regeneration of metal-bearing layered metal sulfides using excess KCl solution, the recycling processes generate a substantial volume of secondary radioactive liquid, which contradicts the waste minimization principle of radiochemistry. Consequently, recycling layered metal sulfides may not present an optimal solution. An alternative approach could involve the solidification of radionuclide-laden layered metal sulfides (Manos and Kanatzidis, 2016). Solidification techniques, such as encapsulation within a matrix or integration into a stable solid matrix, allow for immobilizing the radionuclides in a permanent solid waste form. This strategy guarantees the lasting containment of radioactive materials and curtails the potential release of these materials into the environment. However, there is still a lack of sufficient knowledge and understanding regarding the consolidation of radionuclide-bearing layered metal sulfides into a suitable waste form for long-term disposal. Further research is needed to investigate suitable solidification techniques, evaluate the stability and leaching behavior of the resulting waste form, and ensure compliance with regulatory requirements for long-term disposal.

4. In-depth exploration of layered metal sulfide structures

Most studies have tested the resistance of layered metal sulfides to acid, β, and γ radiation. However, it is regrettable that they all do not further discuss what bestows the layered metal sulfides with the special resistance to acids and radiation. As noted by Gao et al., “far more systematic experiments will be required to make a full assessment of this stability of layered metal sulfides in acid and radiation environment” (Gao et al., 2020). Therefore, there is a pressing need for a more comprehensive and detailed structural characterization of layered metal sulfides to elucidate the reasons behind their resistance to acid and radiation.
